# Effect of Sihogyeji-tang on functional dyspepsia: a systematic review and meta-analysis

**DOI:** 10.3389/fphar.2025.1689132

**Published:** 2025-11-19

**Authors:** Hanbum Bae, Jinsung Kim, Soyeon Kim

**Affiliations:** 1 Department of Korean Medicine, The Graduate School of Pusan National University, Yangsan, Republic of Korea; 2 Department of Gastroenterology, Kyung Hee University College of Korean Medicine, Kyung Hee University Medical Center, Seoul, Republic of Korea; 3 Department of Korean Internal Medicine, Pusan National University Korean Medicine Hospital, Yangsan, Republic of Korea

**Keywords:** Sihogyeji-tang, Chaihu Guizhi decoction, herbal medicine, functional dyspepsia, systematic review, meta-analysis

## Abstract

**Introduction:**

Functional dyspepsia (FD) has a global prevalence of approximately 15% and is characterized by chronic symptoms with an unclear etiology. Herbal medicines, owing to their multifaceted mechanisms, are promising therapeutic options for FD. This study aimed to establish medical evidence for the use of Sihogyeji-tang (SG), a herbal medicine, in the treatment of FD, thereby providing clinically relevant evidence for both patients and healthcare practitioners.

**Methods:**

A comprehensive search was conducted on 24 June 2025, in the following databases—four English databases (CINAHL, EMBASE, Cochrane Database, and PubMed), five Korean databases (RISS, KISS, NDSL, DBPIA, and OASIS), one Japanese database (J-Stage), and three Chinese databases (CNKI, Wanfang, and VIP)—to identify eligible studies for this review. Randomized controlled trials investigating the use of SG for the treatment of FD were included. The risk of bias was assessed using the Cochrane Risk of Bias tool and the results were synthesized with Review Manager 5.4.

**Results:**

Data from 12 randomized controlled trials involving 805 individuals were included in the meta-analysis. SG demonstrated a significantly higher total effective rate than prokinetic agents (risk ratio [RR]: 1.28, 95% confidence interval [CI]: 1.19–1.37, P < 0.00001). SG also resulted in a significantly greater reduction in symptom severity, as measured by the traditional Chinese medicine (TCM) symptom total score, compared with the control group (standardized mean difference: −1.10, 95% CI: −1.53 to −0.68). The incidence of adverse events was significantly lower in the SG group than in the control group (RR: 0.26, 95% CI: 0.09–0.76, P < 0.05). The quality of evidence was rated as moderate for the total effective rate, very low for the TCM symptom score, and low for adverse events.

**Discussion:**

The findings suggest that SG may be more effective and safer than prokinetic agents for FD. However, the certainty of the evidence is limited by methodological weaknesses in the included studies. To validate these results and support the clinical adoption of SG in FD, robust and extensive randomized controlled trials are needed.

**Systematic review registration:**

[https://www.crd.york.ac.uk/PROSPERO/], identifier [CRD420251041781].

## 1 Introduction

Functional dyspepsia (FD) has a global prevalence of approximately 16% (Ford et al., 2020) and is more prevalent in developing countries than in developed countries and among women (Lee et al., 2024). While its prevalence is gradually declining (Lee et al., 2024), FD continues to impair the patients’ quality of life (Enck et al., 2017). It mainly affects the physical components of health-related quality of life rather than the mental components (Hantoro et al., 2018). Diagnosis of FD is based on the Rome IV criteria, which include symptoms, such as early satiety, epigastric burning, postprandial fullness, and epigastric pain for at least 6 months, with no evidence of structural abnormalities (Stanghellini et al., 2016).

The definitive etiology of FD remains unclear; however, gastric dysrhythmias, antral hypomotility, and delayed and rapid gastric emptying have been associated with its pathogenesis (Park et al., 2017). Visceral hypersensitivity—including a lowered threshold for pain with normal gastric compliance, dysfunction of mechanoreceptors, and abnormal afferent signaling in the central nervous system—has also been suggested as a potential mechanism underlying the pathophysiology of FD (Farré et al., 2013; Rosen et al., 2014). In addition, psychological distress, including depression and anxiety, has been linked to FD and, in some individuals, may precede the emergence of symptoms (Aro et al., 2015). Gastroduodenal inflammation or *Helicobacter pylori* infection has also been posited as a cause of FD (Sayuk and Gyawali, 2020; Iwata et al., 2023). Among these, gastric motor dysfunction and visceral hypersensitivity are the most widely accepted causes (Vanheel and Farré, 2013).

Current treatment modalities for FD include acid suppressants and prokinetics (Oshima, 2024). However, because FD involves multiple pathogenic factors, single-target therapies may be insufficient. Consequently, an increasing number of multipotent herbal medicines have been proposed in various countries (Xiao et al., 2022). In Japan, rikkunshito is currently recommended as a first-line therapy for FD in the evidence-based clinical practice guidelines (Miwa et al., 2022).

Sihogyeji-tang (SG), known as Chaihu Guizhi decoction in China and Saiko-keishi-to in Japan, is used to treat depression, influenza, and epilepsy in Asia (Liu et al., 2014; Lee et al., 2016; Zhao et al., 2024). SG is a combination of Sosiho-tang (Xiao Chaihu decoction) and Gyeji-tang (Guizhi decoction) and is composed of *Bupleurum falcatum* L. [Apiaceae; *Bupleuri Radix*], *Scutellaria baicalensis* Georgi [Lamiaceae; *Scutellariae Radix*], *Cinnamomum cassia* (L.) J.Presl [Lauraceae; *Cinnamomi Ramulus*], *Paeonia lactiflora* Pall. [Paeoniaceae; *Paeoniae Radix*], *Panax ginseng* C.A.Mey. [Araliaceae; *Ginseng Radix*], *Pinellia ternata* (Thunb.) Makino [Araceae; *Pinelliae Tuber*], *Glycyrrhiza uralensis* Fisch. ex DC. [Fabaceae; *Glycyrrhizae Radix et Rhizoma*], *Zingiber officinale* Roscoe [Zingiberaceae; *Zingiberis Rhizoma Recens*], and *Ziziphus jujuba* Mill. [Rhamnaceae; *Jujubae Fructus*] (Li et al., 2019). The combination of quercetin and baicalein in Sosiho-tang exhibits anti-inflammatory effects by suppressing the levels of interleukin-6, tumor necrosis factor-α, and interleukin-1β (Zhan et al., 2021; Lei et al., 2024). According to the traditional Korean medicine theory, Sosiho-tang regulates the flow of Qi, strengthens the stomach, promotes blood circulation, and eliminates stasis (Zhan et al., 2021). Gyeji-tang also inhibits proinflammatory cytokines interleukin-6 and tumor necrosis factor-α (Yoo et al., 2016). Pharmacological experimental studies have revealed that Gyeji-tang can play a significant role in the bidirectional regulation of body temperature, sweat glands, immune function, blood pressure, and gastrointestinal motility while also exhibiting antiallergic, anti-inflammatory, antiviral, antibacterial, hypoglycemic, analgesic, and cardiovascular protective effects (Yuan et al., 2017). Given its multifaceted composition, SG has therapeutic potential for FD.

Although multiple randomized controlled trials (RCTs) have evaluated SG for FD, no systematic review has been conducted to summarize and appraise the existing research. Recent meta-analyses have investigated formulas such as rikkunshito (RKT) and Banxia-xiexin-tang (BXT) for FD (Ko et al., 2021; Kim et al., 2023); however, a comprehensive synthesis of SG remains to be performed. As a hybrid of Sosiho-tang and Gyeji-tang, SG represents a distinct therapeutic concept within East Asian gastroenterology, combining anti-inflammatory and motility-modulating actions that may distinguish its clinical efficacy profile. By synthesizing evidence from these clinical trials, we aimed to establish a foundation for the clinical application of SG in FD.

## 2 Methods

### 2.1 Study protocol and registration

The review protocol was registered with PROSPERO (CRD420251041781).

### 2.2 Data sources and search strategy

We systematically searched eleven databases from their inception to June 2025: four English databases (CINAHL, EMBASE, Cochrane Database, and PubMed), five Korean databases (RISS, KISS, NDSL, DBPIA, and OASIS), one Japanese database (J-Stage), and three Chinese databases (Chinese National Knowledge Infrastructure; CNKI, Wanfang Data, and VIP Information; VIP). Two independent researchers performed the search.

The search strategy included terms related to the intervention (e.g., Sihogyeji*, shihogyeji*, Shiho-Guizhi*, Saikokeishito*, Chaihuguizhi*, Chaihu Guizhi*, Modified Chaihuguizhi*, etc.) and the target condition (e.g., “Functional Dyspepsia”, Dyspepsia, “Non-ulcer dyspepsia”, Indigestion, Digest*, Gastr*, Postprandial, Epigastric, Gut, Stomach, Intestin*). Detailed search strategies are presented in Supplementary Appendix A for the English databases and Supplementary Appendix B for the Chinese databases.

No language restrictions were applied, and the search strategies were adapted for each database as appropriate.

### 2.3 Study selection criteria for this review

Two researchers independently selected the data. The selection of studies was performed using Microsoft Excel.

#### 2.3.1 Types of studies

This systematic review included only RCTs. Studies were included regardless of blinding, language, or reporting type.

#### 2.3.2 Types of patients

All patients included in this review were diagnosed with FD, and no criteria other than age (≥18 to ≤70 years) were considered.

#### 2.3.3 Types of interventions

Studies using SG or modified SG as the sole herbal intervention were included. To objectively define “modified SG”, we established criteria based on the traditional principles of formula composition, which describe the hierarchical roles of herbs as the Chief (君, jūn), Deputy (臣, chén), Assistant (佐, zuǒ), and Envoy (使, shǐ) (Bensky and Barolet, 1990).

The therapeutic integrity of a formula is primarily determined by its Chief and Deputy herbs, which target the main clinical presentation. Therefore, our primary inclusion criterion was the mandatory presence of the six core herbs that function as the Chief and Deputy components of SG: *Bupleurum falcatum* L. [Apiaceae; *Bupleuri Radix*], *Scutellaria baicalensis* Georgi [Lamiaceae; *Scutellariae Radix*], *Cinnamomum cassia* (L.) J.Presl [Lauraceae; *Cinnamomi Ramulus*], *Paeonia lactiflora* Pall. [Paeoniaceae; *Paeoniae Radix*], *Panax ginseng* C.A.Mey. [Araliaceae; *Ginseng Radix*] (or its common substitute, *Codonopsis pilosula* (Franch.) Nannf. [Campanulaceae; *Codonopsis Radix*]), and *Pinellia ternata* (Thunb.) Makino [Araceae; *Pinelliae Tuber*].

Based on this core structure, the following modifications were permitted:

1. Omission: The omission of up to three herbs serving as Assistant and Envoy herbs, which primarily harmonize the formula, was allowed. These include *Glycyrrhiza uralensis* Fisch. ex DC. [Fabaceae; *Glycyrrhizae Radix et Rhizoma*], *Zingiber officinale* Roscoe [Zingiberaceae; *Zingiberis Rhizoma Recens*], and *Ziziphus jujuba* Mill. [Rhamnaceae; *Jujubae Fructus*].
2. Addition: The addition of a maximum of five herbs based on syndrome differentiation (辨證加減, biànzhèng jiājiǎn) was permitted.

All the included studies administered the formula orally, regardless of its form (e.g., decoctions, powders). For the control group, studies using standard pharmacologic treatments (e.g., prokinetics, proton pump inhibitors) or usual care for FD were eligible. However, interventions combining SG with other treatments not specified in the control group were excluded.

#### 2.3.4 Types of outcome measures

The outcomes analyzed in this review were as follows:

Primary outcome:

1. Total effective rate: This outcome reflects the percentage of patients who experienced improvement following treatment, where improvement was defined as any response except “ineffective.” As this measure is widely used to indicate treatment effectiveness in Chinese RCTs, it was adopted as an outcome in this review.

Secondary outcomes:

1. TCM symptom total score: This outcome refers to the sum of scores for various symptoms (such as epigastric pain, poor appetite, epigastric discomfort, and other related symptoms) based on traditional Chinese medicine diagnostic criteria. Higher scores indicate more severe symptoms. This measure is commonly used in Chinese clinical trials to quantitatively assess changes in symptom severity before and after treatment.
2. Adverse events: The outcome was determined by the rate of participants who encountered any adverse event at any point during or after treatment. Adverse events were recorded to assess the safety profile of the interventions.

### 2.4 Data extraction and analysis

#### 2.4.1 Data extraction

For each included study, two researchers independently extracted data as planned. The following data items were collected: publication year, first author, study design, country, sample size and characteristics (sex, age, diagnostic criteria), intervention details, outcome measures, any reported adverse events, and blinding methods. Discrepancies were resolved by discussion. All extracted data were managed using Microsoft Excel.

#### 2.4.2 Assessment of risk of bias

Two reviewers independently evaluated the risk of bias in each study using the Cochrane Risk of Bias tool, following the criteria outlined in the Cochrane Handbook (Higgins et al., 2011). The following domains were included: random sequence generation, allocation concealment, blinding of both participants and personnel, incomplete outcome data, selective reporting, and other potential biases. Each category was judged as low, unclear, or high risk. Differences in judgment were resolved through discussion.

#### 2.4.3 Data synthesis

Review Manager (RevMan) version 5.4 (Cochrane) was employed for all statistical analyses. Risk ratios were utilized to analyze dichotomous outcomes, whereas standardized mean differences were employed to assess continuous outcomes, both reported with 95% confidence intervals (CIs).

#### 2.4.4 Dealing with missing data

If important data were missing or unclear, we contacted the authors of the studies to seek clarification. Studies were excluded from the analysis if the needed data remained unobtainable.

#### 2.4.5 Assessment of heterogeneity

The Chi-square test and I^2^ statistic were utilized to evaluate heterogeneity among the included studies. Sensitivity and subgroup analyses were planned to explore potential sources of heterogeneity if substantial heterogeneity was detected (I^2^ ≥ 50%) and if a sufficient number of studies (≥10) was available. A fixed-effect model would be used if heterogeneity was low (I^2^ < 50%), whereas a random-effects model was planned for high heterogeneity (I^2^ ≥ 50%).

#### 2.4.6 Assessment of reporting bias

Reporting bias assessment was intended to be conducted using funnel plots. This analysis would be conducted only if the meta-analysis included at least 10 studies.

#### 2.4.7 Grading the quality of evidence

The quality of evidence was independently assessed by two researchers. The certainty of evidence for the outcomes was assessed using the GRADE approach, implemented via the GRADEpro Guideline Development Tool (https://gradepro.org/). The evaluation considered the following domains: study design, risk of bias, inconsistency, indirectness, imprecision, and other relevant factors. Following these assessments, the quality of evidence was rated as high, moderate, low, or very low (Table 1).

**TABLE 1:** Quality of evidence.

	Certainty assessment							No of patients		Effect		Certainty
	No of studies	Study design	Risk of bias	Inconsistency	Indirectness	Imprecision	Other considerations	SG	WM	Relative (95% CI)	Absolute (95% CI)	
Total effective rate	12	Randomized trials	Serious^a^	Not serious	Not serious	Not serious	None	368/403 (91.3%)	287/402 (71.4%)	RR 1.28 (1.19–1.37)	200 more per 1,000 (from 136 more to 264 more)	⨁⨁⨁◯ Moderate
TCM symptom total score	4	Randomized trials	Serious^a^	Serious^b^	Not serious	Serious^c^	None	128	128	-	SMD 1.1 lower (1.53 lower to 0.68 lower)	⨁◯◯◯ Very low
Adverse events	4	Randomized trials	Serious^a^	Not serious	Not serious	Serious^c^	None	4/115 (3.5%)	15/114 (13.2%)	RR 0.26 (0.09–0.76)	97 fewer per 1,000 (from 120 fewer to 32 fewer)	⨁⨁◯◯ Low

SG, Sihogyeji-tang group; WM, western medication group; CI, confidence interval; RR, risk ratio; SMD, standardized mean difference; TCM, traditional chinese medicine.

^a^ ROB, was not low.

^b^ Substantial statistical heterogeneity (I^2^ = 58%).

^c^ Sample size was not sufficient.

### 2.5 Ethical approval

No ethical approval was required as only published data were analyzed.

## 3 Results

### 3.1 Study selection

**FIGURE 1:**
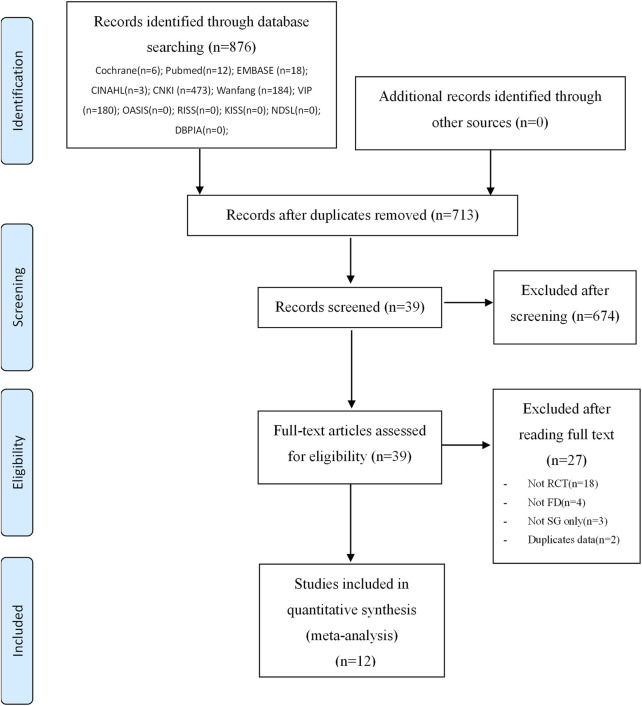
PRISMA flowchart of the literature search process. Cochrane, Cochrane Central Register of Controlled Trials; PubMed, National Library of Medicine biomedical literature database; EMBASE, Excerpta Medica database for biomedical and pharmaceutical literature; CINAHL, Cumulative Index to Nursing and Allied Health Literature; CNKI, China National Knowledge Infrastructure; Wanfang, Wanfang Data; VIP, VIP Information; OASIS, Oriental Medicine Advanced Searching Integrated System; RISS, Research Information Service System; KISS, Korean Studies Information Service System; NDSL, National Digital Science Library; DBPIA, academic journal database; J-Stage, Japan Science Technology Information Aggregator, Electronic.

A total of 876 studies were initially identified, and 713 remained after removing duplicates. After screening titles and abstracts, 39 studies were selected. Upon full-text assessment of these 39 studies, 27 were excluded for various reasons: 18 were not RCTs, 4 did not study FD, 3 did not include SG monotherapy as the intervention (e.g., combination therapy with the control drug), and 2 presented duplicate experimental data. Consequently, 12 studies were selected for inclusion in our meta-analysis. The study selection procedure is depicted in the PRISMA flow chart (Figure 1).

### 3.2 Participants

The 12 studies comprised a total of 805 participants, all of whom were aged 18–70 years.

### 3.3 Intervention

#### 3.3.1 Experimental intervention

In all the 12 studies (Li, 2011; Chen, 2014; Li, 2016; Wang, 2017; Dai, 2019; Deng and Dong, 2019; Yu, 2019; Su, 2020; Zhang, 2020; Huang, 2021; Shao, 2021; Li, 2022) included in the meta-analysis, SG or modified SG was administered as an oral decoction. Five studies (Li, 2016; Dai, 2019; Su, 2020; Zhang, 2020; Li, 2022) administered the intervention for 30 days, whereas the remaining studies administered it for 28 days. In all studies, SG was administered twice daily (Table 2).

**TABLE 2:** Characteristics of included studies.

First Author (Year)	Sample Age (y), sex (m/f)	Intervention (experimental)	Control	Outcomes	Result
Li (2022) RCT	Age A: 49.15 ± 5.10 (24–68) B: 48.65 ± 5.15 (24–67) Sex A: 17/10 B: 18/8	(A) SG, 100 mL (bid, total 30 days): Bupleuri radix 30g, Cinnamomi ramulus 15g, Scutellariae radix 20g, Codonopsis Pilosulae radix 15g, Paeoniae Radix 12g, Pinelliae tuber preparatum 12g, Glycyrrhizae radix praeparata 10g, Zizyphi fructus 10g, Zingiberis rhizoma recens 10 g Extraction: Add 400 mL of water and boil down to 200 mL	(B) Western medication (mosapride Citrate tablets 5 mg, orally, three times a day, taken with warm water, for 30 continuous days)	1. Total effective rate 2. Sleep quality (PSQI: Subjective sleep quality, sleep latency, sleep duration, sleep efficiency, use of sleep medication, daytime dysfunction) 3. Adverse events (diarrhea, dizziness, rash)	1. RR 1.25 [1.00, 1.56], P > 0.05. 2. Subjective sleep quality, SMD -3.56 [-5.12, −1.99], P < 0.05; Sleep latency, SMD -3.52 [-5.07, −1.96], P < 0.05; Sleep duration, SMD -2.98 [-4.39, −1.57], P < 0.05; Sleep efficiency, SMD -3.53 [-5.08, −1.98], P < 0.05; use of sleep medication, SMD -4.03 [-5.62, −2.44], P < 0.05; Daytime dysfunction, SMD -4.26 [-6.03, −2.48], P > 0.05 3. RR 0.12 [0.02, 0.90], P < 0.05
Shao (2021) RCT	Age A: 40.5 ± 2.7 (22–54) B: 40.3 ± 2.8 (21–53) Sex A: 20/15 B: 22/13	(A) SG, not reported (bid, total 28 days) Cinnamomi ramulus 15g, Scutellariae radix 9g, Bupleuri radix 12g, Paeoniae radix alba 15g, Codonopsis Pilosulae radix 15g, Carthami Flos 6g, Pinelliae tuber preparatum 10g, Glycyrrhizae radix praeparata 10g, Trionycis Carapax 6g, Ostreae Concha 15g, Rubiae Radix et Rhizoma 10g, Zingiberis rhizoma recens 10 g Extraction: Water	(B) Western medication (mosapride Citrate tablets 5 mg, orally, three times a day, for 4 continuous weeks)	1. Total effective rate 2. Gastric emptying rate 3. Symptom relief time (abdominal distension, diarrhea, abdominal pain)	1. RR 1.27 [1.03, 1.57], P < 0.05 2. SMD 3.08 [2.38, 3.79], P < 0.00001 3. Abdominal distension, SMD -1.20 [-1.71, −0.69], P < 0.00001; Diarrhea, SMD -1.03 [-1.53, −0.53], P < 0.0001; Abdominal pain, SMD -1.50 [-2.03, −0.96], P < 0.00001
Huang (2021) RCT	Age A: 38.49 ± 12.64 B: 37.52 ± 12.73 Sex A: 15/18 B: 16/17	(A) SG, 200 mL (bid, total 28 days) Bupleuri radix 20g, Scutellariae radix 15g, Pinelliae tuber preparatum 15g, Codonopsis Pilosulae radix 20g, Zingiberis rhizoma recens 10g, Glycyrrhizae radix praeparata 10g, Cinnamomi ramulus 20g, Paeoniae radix alba 20g, Zizyphi fructus 10 g Extraction: The decoction was prepared into 200mL/bag standard preparations by the pharmacy of Ruikang Hospital affiliated to Guangxi University of Chinese medicine	(B) Western medication (domperidone tablets 1 tablet and compound Azintamide enteric-coated tablets 2 tablets, orally, three times a day, for 4 continuous weeks)	1. Total effective rate 2. Gastrointestinal symptoms score (postprandial fullness, early satiety) 3. TCM symptoms score (epigastric distension, epigastric pain, aggravated by cold, dry mouth or bitter taste, poor appetite, stomach discomfort, nausea/vomiting, borborygmus, loose stool) 4. TCM symptom total score 5. Anxiety (HAMA) and depression (HAMD) scores 6. Recurrence rate 7. Adverse events (diarrhea, insomnia)	1. RR 1.30 [1.02, 1.67], P < 0.05. 2. Postprandial fullness, SMD -0.17 [-0.65, 0.32], P > 0.05; early satiety, SMD -0.35 [-0.84, 0.13], P > 0.05. 3. Epigastric distension, SMD -0.53 [-1.01, −0.04], P < 0.05; epigastric pain, SMD -0.30 [-0.78, 0.18], P > 0.05; Aggravated by cold, SMD -0.83 [-1.34, −0.33], P < 0.01; Dry mouth or bitter taste, SMD -0.52 [-1.01, −0.03], P < 0.05; poor appetite, SMD -0.68 [-1.17, −0.18], P < 0.01; stomach discomfort, SMD -0.57 [-1.07, −0.08], P < 0.05; Nausea/vomiting, SMD -0.26 [-0.74, 0.23], P > 0.05; Borborygmus, SMD -0.43 [-0.91, 0.06], P > 0.05; Loose stool, SMD -0.88 [-1.38, −0.37], P < 0.001 4. SMD -1.05 [-1.56, −0.53], P < 0.0001 5. HAMA, SMD -1.16 [-1.67, −0.62], P < 0.0001; HAMD, SMD -1.14 [-1.66, −0.62], P < 0.0001. 6. RR 0.33 [0.10, 1.13], P > 0.05. 7. RR 1.00 [0.07, 15.33], P > 0.05
Zhang (2020) RCT	Age A: 45.50 ± 6.70 (21–69) B: 45.65 ± 6.50 (20–68) Sex A: 31/19 B: 29/21	(A) SG, 200 mL (bid, total 30 days) Cinnamomi ramulus 15g, Codonopsis Pilosulae radix 15g, Trionycis Carapax 20g, Ostreae Concha 15g, Bupleuri radix 12g, Paeoniae radix alba 15g, Pinelliae tuber preparatum 10g, Scutellariae radix 9g, Eupolyphaga seu Steleophaga 6g, Rubiae Radix et Rhizoma 10g, Glycyrrhizae radix praeparata 10g, Carthami Flos 6g, Zingiberis rhizoma recens 10 g Extraction: Soak the daily dose in 800 mL of water for 30 min, boil, then simmer for 30 min to get 300 mL of liquid. Add 500 mL of water again, boil, then simmer for 30 min to get 200 mL of liquid. Mix the two extracts to make 400 mL	(B) Western medication (mosapride Citrate tablets 5 mg, orally, three times a day, for 30 continuous days)	1. Total effective rate 2. TCM symptom total score	1. RR 1.24 [1.04, 1.47], P < 0.05 2. SMD -0.65 [-1.05, −0.25], P < 0.01
Su (2020) RCT	Age A: 45.32 ± 2.36 (25–65) B: 47.23 ± 2.12 (23–67) Sex A: 6/4 B: 5/5	(A) SG, 250 mL (bid, total 30 days) Codonopsis Pilosulae radix 20g, Ostreae Concha 20g, Cinnamomi ramulus 10g, Bupleuri radix 15g, Scutellariae radix 9g, Pinelliae tuber preparatum 9g, Paeoniae radix alba 15g, Atractylodis rhizoma alba 10g, Glycyrrhizae radix praeparata 9g, Zingiberis rhizoma recens 9 g Extraction: Water. Boil to make 500 mL	(B) Western medication (domperidone tablets 10 mg, orally, three times a day, 30 min before meals, for 30 continuous days)	1. Total effective rate	1. RR 1.40 [0.92, 2.14], P > 0.05
Dai (2019) RCT	Age A: 52.9 ± 4.7 (34–69) B: 53.1 ± 5.5 (36–68) Sex A: 24/24 B: 23/25	(A) SG, 100 mL (bid, total 30 days): Bupleuri radix 16g, Scutellariae radix 10g, Pinelliae tuber 10g, Zingiberis rhizoma 10g, Codonopsis Pilosulae radix 8g, Glycyrrhizae radix praeparata 8g, Cinnamomi ramulus 12g, Paeoniae radix alba 12g, Zizyphi fructus 12g, Curcumae Longae Rhizoma 12 g Extraction: Soak the daily dose in 1500 mL of water for 30 min, then boil down to 200 mL	(B) Western medication (mosapride Citrate tablets 5 mg, orally, three times a day, for 30 continuous days)	1. Total effective rate 2. Symptom relief time (abdominal distension, diarrhea, abdominal pain)	1. RR 1.28 [1.07, 1.52], P < 0.01 2. Abdominal distension, SMD -1.11 [-1.54, −0.68], P < 0.00001; Diarrhea, SMD -1.65 [-2.12, −1.19], P < 0.00001; Abdominal pain, SMD -1.74 [-2.21, −1.27], P < 0.00001
Deng and Dong (2019) RCT	Age A: 41.73 ± 3.61 (22–58) B: 41.25 ± 3.64 (21–59) Sex A: 17/8 B: 15/10	(A) SG, 200 mL (bid, total 28 days): Bupleuri radix 12g, Pinelliae tuber preparatum 10g, Codonopsis Pilosulae radix 15g, Rubiae Radix et Rhizoma 10g, Zingiberis rhizoma recens 10g, Trionycis Carapax 20g, Carthami Flos 6g, Ostreae Concha 15g, Glycyrrhizae radix praeparata 10g, Scutellariae radix 9g, Cinnamomi ramulus 15g, Paeoniae radix alba 15g, Eupolyphaga seu Steleophaga 6 g Extraction: Soak the daily dose in 800 mL of water for 30 min, boil, then simmer for 30 min to get 300 mL of liquid. Add 500 mL of water again, boil, then simmer for 30 min to get 200 mL of liquid. Mix the two extracts to make 400 mL	(B) Western medication (mosapride Citrate tablets 5 mg, orally, three times a day, for 4 continuous weeks)	1. Total effective rate 2. TCM symptom total score	1. RR 1.35 [1.01, 1.81], P < 0.05. 2. SMD -1.34 [-1.96, −0.72], P < 0.0001
Yu (2019) RCT	Age (20–57) A: 34.25 ± 10.89 B: 37.80 ± 11.54 Sex A: 8/12 B: 13/7	(A) SG, 100 mL (bid, total 28 days): Bupleuri radix 25g, Cinnamomi ramulus 15g, Pinelliae tuber preparatum 12g, Paeoniae Radix 15g, Scutellariae radix 20g, Codonopsis Pilosulae radix 15g, Glycyrrhizae radix praeparata 10g, Zingiberis rhizoma recens 10g, Zizyphi fructus 10 g Extraction: Water. Boil to make 200 mL	(B) Western medication (mosapride Citrate tablets 5 mg, orally, three times a day, for 4 continuous weeks)	1. Total effective rate 2. Gastrointestinal symptoms score (postprandial fullness, early satiety, epigastric pain, epigastric burning) 3. Total gastrointestinal symptom score 4. TCM symptoms score (epigastric distension/pain, aggravated by cold, dry mouth or bitter taste, poor appetite, stomach discomfort, nausea/vomiting, borborygmus, loose stool) 5. TCM symptom total score 6. Serum 5-HT level (ng/mL) 7. Anxiety (HAMA) and depression (HAMD) scores 8. Recurrence rate 9. Adverse events	1. RR 1.46 [1.04, 2.05], P < 0.05 2. Postprandial fullness, SMD -0.66 [-1.30, −0.03], P < 0.05; early satiety, SMD -0.16 [-0.78, 0.46], P > 0.05; epigastric pain, SMD -0.72 [-1.36, −0.08], P < 0.05; epigastric burning, SMD -0.08 [-0.70, 0.54], P > 0.05 3. SMD -1.10 [-2.01, −0.19], P < 0.05 4. Epigastric distension or pain, SMD -0.72 [-1.36, −0.08], P < 0.05; Aggravated by cold, SMD -1.23 [-1.92, −0.55], P < 0.001; Dry mouth or bitter taste, SMD -0.05 [-0.57, 0.67], P > 0.05; poor appetite, SMD -0.67 [-1.31, −0.03], P < 0.05; stomach discomfort, SMD -0.65 [-1.29, −0.02], P < 0.05; Nausea/vomiting, SMD -0.29 [-0.91, 0.33], P > 0.05; Borborygmus, SMD -0.19 [-0.81, 0.43], P > 0.05; Loose stool, SMD -0.10 [-0.72, 0.52], P > 0.05 5. SMD -1.65 [-2.37, −0.92], P < 0.0001 6. SMD 2.33 [1.53, 3.13], P < 0.05 7. HAMA, SMD -1.69 [-2.43, −0.96], P < 0.00001; HAMD, SMD -1.56 [-2.28, −0.85], P < 0.0001 8. RR 0.41 [0.12, 1.43], P > 0.05. 9. A: 0% B: 0%, P = 1
Wang (2017) RCT	Age: 19–67 Sex: 47/33	(A) SG, 200 mL (bid, total 28 days): Cinnamomi ramulus 15g, Codonopsis Pilosulae radix 15g, Trionycis Carapax 20g, Ostreae Concha 15g, Bupleuri radix 12g, Paeoniae radix alba 15g, Pinelliae tuber preparatum 10g, Scutellariae radix 9g, Eupolyphaga seu Steleophaga 6g, Rubiae Radix et Rhizoma 10g, Glycyrrhizae radix praeparata 10g, Carthami Flos 6g, Zingiberis rhizoma recens 10 g Extraction: Soak the daily dose in 800 mL of water for 30 min, boil, then simmer for 30 min to get 300 mL of liquid. Add 500 mL of water again, boil, then simmer for 30 min to get 200 mL of liquid. Mix the two extracts to make 400 mL	(B) Western medication (mosapride Citrate tablets 5 mg, orally, three times a day, for 4 continuous weeks)	1. Total effective rate 2. Gastric emptying rate	1. RR 1.23 [1.01, 1.51], P < 0.05 2. SMD 0.94 [0.48, 1.40], P < 0.05
Li (2016) RCT	Age A: 18–63 B: 22–69 Sex A: 20/20 B: 17/23	(A) SG, not reported (bid, total 30 days): Bupleuri radix 10g, Codonopsis Pilosulae radix 11g, Pinelliae tuber preparatum 9g, Zingiberis rhizoma recens 6g, 6 Zizyphi fructus, Glycyrrhizae radix praeparata 10g, Cinnamomi ramulus 9g, Paeoniae radix alba 10 g (B) Extraction: Water.	(B) Western medication (Itopride Hydrochloride tablets, orally, three times a day, 30 min before meals, for 30 continuous days)	1. Total effective rate 2. Dyspepsia improvement rate	1. RR 1.32 [0.94, 1.85], P > 0.05 2. RR 1.92 [1.16, 3.19], P < 0.05
Chen (2014) RCT	Age A: 52 ± 5.92 (36–67) B: 53.5 ± 2.96 (37–69) Sex A: 19/16 B: 20/15	(A) SG, 100 mL (bid, total 28 days): Bupleuri radix 10g, Glycyrrhizae radix praeparata 10g, Zingiberis rhizoma recens 6g, Cinnamomi ramulus 8g, Scutellariae radix 6g, Codonopsis Pilosulae radix 10g, Pinelliae tuber preparatum 9g, Paeoniae radix alba 12g, 6 Zizyphi fructus Extraction: Water. Boil to make 200 mL	(B) Western medication (domperidone tablets 10 mg, orally, three times a day, 30 min before meals, for 4 continuous weeks)	1. Total effective rate 2. Adverse events (diarrhea, dizziness, headache)	1. RR 1.19 [0.96, 1.46], P > 0.05. 2. RR 0.33 [0.07, 1.54], P > 0.05
Li (2011) RCT	Age A: 47 (18–68) B: 46 (21–70) Sex A: 16/24 B: 14/26	(A) SG, 100 mL (bid, total 28 days): Bupleuri radix 10g, Scutellariae radix 6g, Pinelliae tuber preparatum 9g, Codonopsis Pilosulae radix 10g, Glycyrrhizae radix praeparata 6g, Cinnamomi ramulus 8g, Paeoniae radix alba 12g, Zingiberis rhizoma recens 6g, 6 Zizyphi fructus. Extraction: Water. Boil to make 200 mL	(B) Western medication (domperidone tablets 10 mg, orally, three times a day, 30 min before meals, for 4 continuous weeks)	1. Total effective rate 2. Single symptom total effective rate	1. RR 1.29 [1.02, 1.61], P < 0.05 2. RR 1.19 [0.99, 1.44], P > 0.05

SG, Sihogyeji-tang; PSQI, pittsburgh sleep quality index; TCM, traditional chinese medicine; 5-HT, 5-hydroxytryptamine; HAMA, hamilton anxiety rating scale; HAMD, hamilton depression rating scale; RR, risk ratio; SMD, standardized mean difference; RCT, randomized controlled trial.

#### 3.3.2 Control intervention

Only studies with prokinetic agents as comparators were included. In all studies, an oral prokinetic agent was used as Western medication (WM); three studies (Li, 2011; Chen, 2014; Su, 2020) used Domperidone Tablets, whereas seven studies (Wang, 2017; Dai, 2019; Deng and Dong, 2019; Yu, 2019; Zhang, 2020; Shao, 2021; Li, 2022) used Mosapride Citrate Tablets. One study (Huang, 2021) administered Domperidone Tablets plus Azintamide Tablets, and one study (Li, 2016) used Itopride Tablets. The medications were administered three times daily, and the treatment duration was identical to that of the experimental group (Table 2).

### 3.4 Outcomes

All the 12 studies assessed the total effective rate. Four studies (Deng and Dong, 2019; Yu, 2019; Zhang, 2020; Huang, 2021) evaluated the TCM symptom total score, and four studies (Chen, 2014; Yu, 2019; Huang, 2021; Li, 2022) reported adverse events (Table 2).

### 3.5 Risk of bias

**FIGURE 2:**
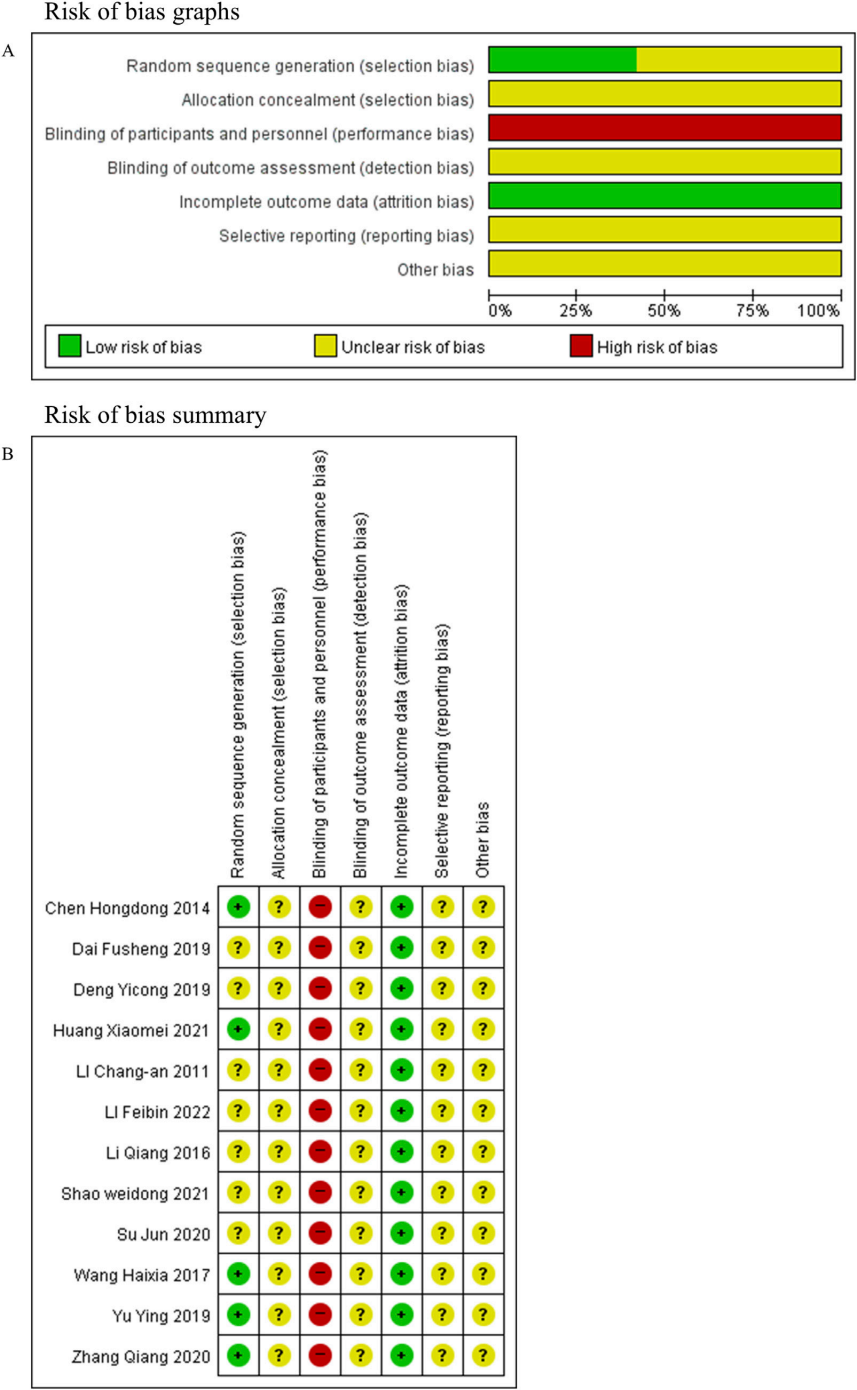
Risk of bias. **(A)** Risk of bias graph. **(B)** Risk of bias summary.

Each of the 12 studies included reports of random sequence generation. Nevertheless, only five (Chen, 2014; Wang, 2017; Yu, 2019; Zhang, 2020; Huang, 2021) studies provided specific details regarding their randomization methods. None reported allocation concealment, outcome assessment blinding, or selective reporting. Blinding of participants and personnel was assessed as high risk of bias in all studies, as the interventions compared oral decoctions with tablets, without placebo control, making blinding of both participants and investigators infeasible. No study had missing outcome data. The risk of bias evaluation results are presented in Figures 2A,B.

### 3.6 Effect of interventions

#### 3.6.1 Total effective rate

**FIGURE 3:**
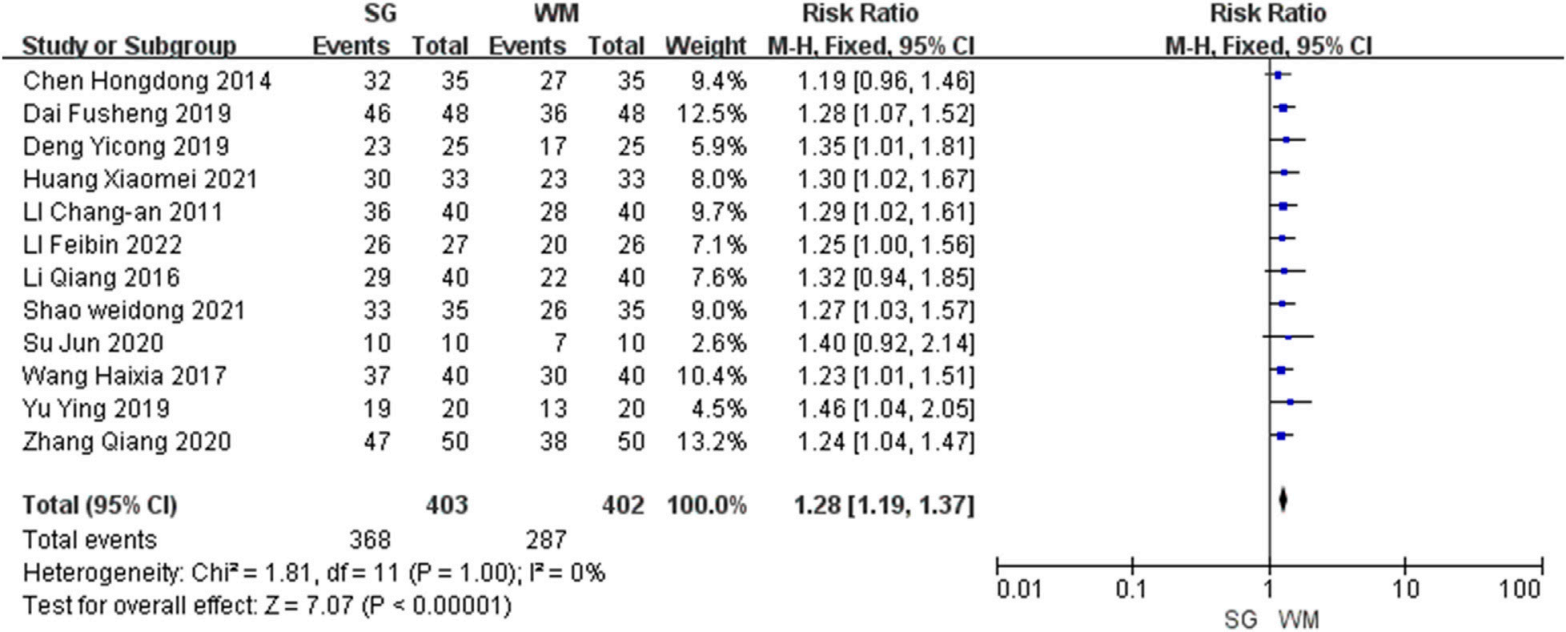
Forest plot of total effective rates comparing Sihogyeji-tang with Western medication.

All the 12 studies analyzed the total effective rate, with a combined total of 805 participants. Among these studies, eight (Li, 2011; Wang, 2017; Dai, 2019; Deng and Dong, 2019; Yu, 2019; Zhang, 2020; Huang, 2021; Shao, 2021) reported significant differences (P < 0.05), whereas the remaining four (Chen, 2014; Li, 2016; Su, 2020; Li, 2022) found no significant differences (P ≥ 0.05). The meta-analysis demonstrated that the total effective rate was significantly higher in the SG group than in the WM group (risk ratio: 1.28, 95% CI: 1.19–1.37, P < 0.00001; Figure 3).

#### 3.6.2 TCM symptom total score

**FIGURE 4:**
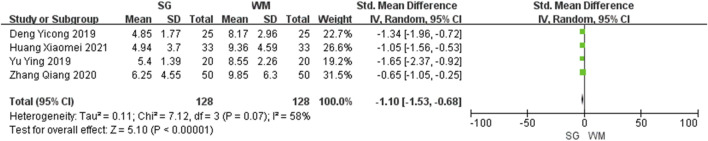
Forest plot of traditional Chinese medicine symptom total score comparing Sihogyeji-tang with Western medication.

Four studies (Deng and Dong, 2019; Yu, 2019; Zhang, 2020; Huang, 2021), comprising a total of 256 participants, reported the TCM symptom total score. All the four studies found significant differences (P < 0.01). The meta-analysis showed that the SG group achieved a significantly greater reduction in the TCM symptom total score than the WM group (standardized mean difference: −1.10, 95% CI: −1.53 to −0.68; Figure 4).

#### 3.6.3 Adverse events

**FIGURE 5:**
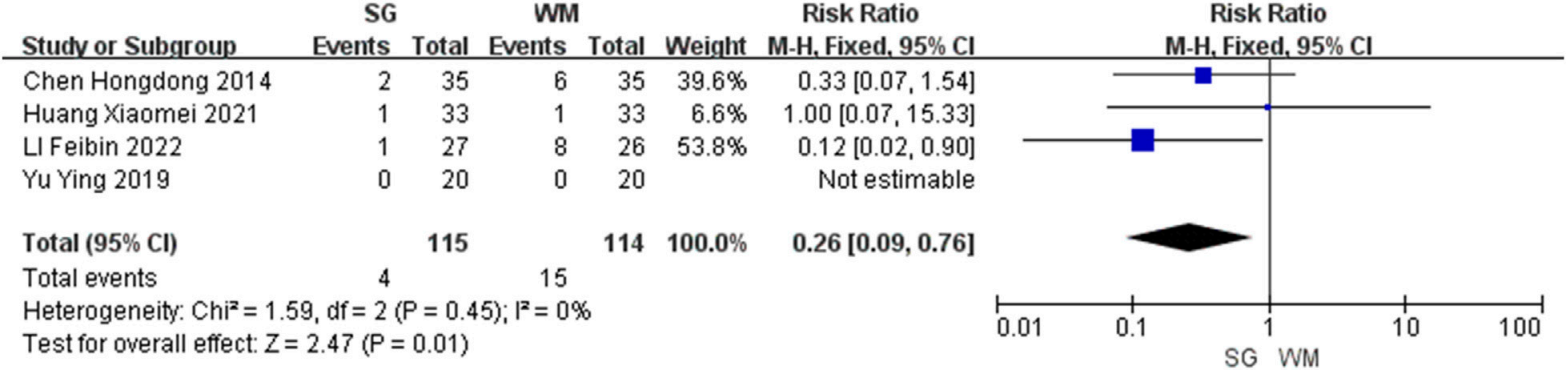
Forest plot of adverse events comparing Sihogyeji-tang with Western medication.

Four studies (Chen, 2014; Yu, 2019; Huang, 2021; Li, 2022), including a total of 229 participants, reported adverse events. Among these, one study (Li, 2022) found a significant difference (P < 0.05), two (Chen, 2014; Huang, 2021) found no significant difference (P ≥ 0.05), and the other (Yu, 2019) reported no adverse events in either group (P = 1). The meta-analysis indicated that the SG group experienced significantly fewer adverse events than the WM group (risk ratio: 0.26, 95% CI: 0.09–0.76, P < 0.05; Figure 5). No serious adverse events were reported.

### 3.7 Heterogeneity

The I^2^ values for the total effective rate and adverse events were <50%, indicating low heterogeneity. Therefore, for these outcomes, sensitivity and subgroup analyses were not performed, and a fixed-effect model was employed for the meta-analyses.

In contrast, the I^2^ value for the TCM symptom total score was 58%, which exceeds the threshold for substantial heterogeneity (I^2^ > 50%). Therefore, a random-effects model was employed for this meta-analysis. Sensitivity and subgroup analyses were not performed because the number of studies (N = 4) did not meet the pre-specified requirement of ≥10 studies.

### 3.8 Reporting bias

**FIGURE 6:**
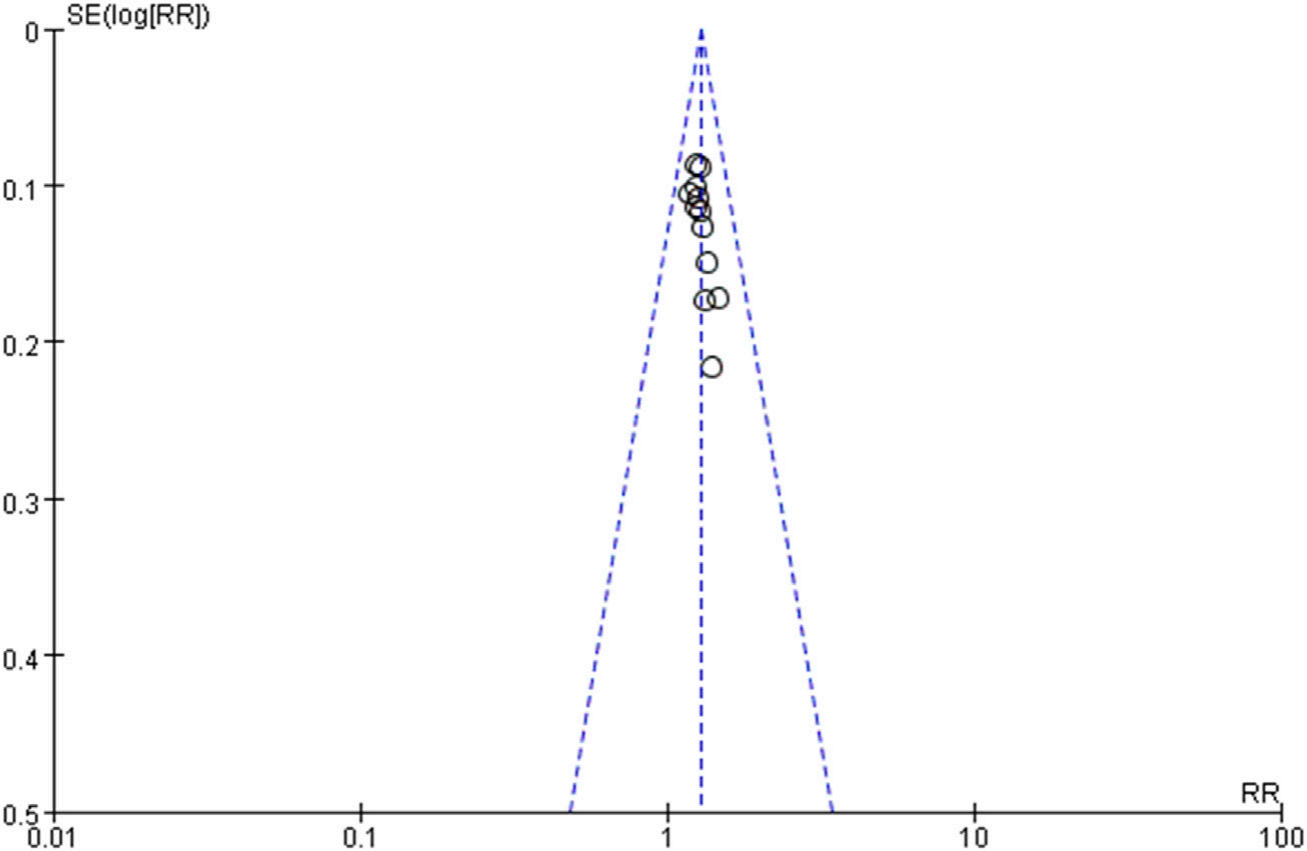
Funnel plot of 12 studies evaluating total effective rate.

Reporting bias assessment was conducted for analyses including ≥10 studies; accordingly, it was performed for the meta-analysis of the total effective rate, which included 12 studies. The funnel plot (Figure 6) revealed a largely symmetrical distribution of studies, suggesting a low risk of publication bias.

### 3.9 GRADE assessment for quality of evidence

The quality of evidence for all the three outcomes was not rated as high, primarily because of risk of bias concerns. The quality of evidence for the TCM symptom total score was also downgraded for inconsistency due to substantial statistical heterogeneity. In addition, owing to limited sample sizes, the evidence quality for both the TCM symptom total score and adverse events was downgraded for imprecision. As a result, the quality of evidence was rated as moderate for the total effective rate, very low for the TCM symptom total score, and low for adverse events (Table 1).

## 4 Discussion

This systematic review and meta-analysis evaluated the efficacy and safety of SG compared with prokinetic agents in the management of FD. The findings indicate that SG is associated with a significantly higher total effective rate and greater improvement in symptom severity, as measured by the TCM symptom total score, than conventional prokinetic therapy. Furthermore, SG showed a better safety profile, exhibiting fewer adverse events.

The present findings, demonstrating that SG provides clinically meaningful symptom relief often with fewer adverse effects than conventional pharmacotherapy, are consistent with those of previous meta-analyses on other multi-component herbal formulations for FD (Gwee et al., 2021; Luo et al., 2022). Herbal medicines, including SG, are believed to exert their effects through multiple mechanisms, such as modulating gastrointestinal motility, reducing inflammation, and neuromodulation, thereby addressing the multifactorial pathophysiology of FD more comprehensively than single-target drugs (Gwee et al., 2021; Luo et al., 2022).

The pharmacological actions of SG are believed to emanate from synergistic interactions among its various herbal constituents. The main active compounds of SG include flavonoids (such as baicalin, isoliquiritigenin, and wogonoside), triterpenoids (such as saikosaponin A and glycyrrhizic acid), and organic acids (such as gallic acid) (Li et al., 2019). These compounds have demonstrated a range of therapeutic effects, including anti-inflammation, anti-oxidation, regulation of blood lipids, vasodilation, and inhibition of platelet aggregation (Li et al., 2019). For instance, baicalin reduces acute inflammation by downregulating the expression of nuclear factor kappa B proteins and protein kinase D1 (Qian et al., 2018), whereas wogonoside reduces inflammation by inhibiting both the nuclear factor kappa B signaling pathway and the activation of the NLRP3 inflammasome (Sun et al., 2015). Saikosaponin A has been shown to inhibit nuclear factor kappa B activation and suppress pro-inflammatory cytokines (Lu et al., 2012). In addition, accumulating evidence indicates that these bioactive compounds of SG also play an important role in regulating gastrointestinal motility, a key pathophysiological mechanism of FD. For example, saikosaponin D, a principal component of Bupleurum species, has been shown in animal studies to improve gastrointestinal motility and gastric emptying by restoring interstitial cells of Cajal and modulating gastrointestinal hormones, such as ghrelin and substance P (Zeng et al., 2024). Furthermore, isoliquiritigenin modulates gastrointestinal motility bidirectionally, inhibiting it at low doses and stimulating it at high doses (Chen et al., 2009). Such multi-targeted actions, addressing various underlying factors, may contribute significantly to the observed clinical efficacy of SG in FD.

Previous meta-analyses on herbal medicines for FD have primarily evaluated formulas such as RKT and BXT, each showing efficacy compared with conventional therapy but differing in pharmacological emphasis—prokinetic, gastroprotective, or psychotropic (Ko et al., 2021; Kim et al., 2023). In contrast, SG integrates two classical prescriptions (Sosiho-tang and Gyeji-tang) that jointly target gastrointestinal motility and inflammatory regulation, offering a dual mechanism distinct from that of other herbal formulas. The present meta-analysis quantitatively demonstrates that SG (risk ratio = 1.28 [1.19–1.37]) achieves a therapeutic effect comparable with or slightly superior to those of RKT (risk ratio = 1.21 [1.17–1.25]) and BXT (risk ratio = 1.19 [1.15–1.23]) (Ko et al., 2021; Kim et al., 2023). Furthermore, the incidence of adverse events with SG (risk ratio = 0.26 [0.09–0.76]) was notably lower than that reported for BXT (risk ratio = 0.53 [0.35–0.81]) and significantly lower compared with RKT, for which the previous meta-analysis found no significant difference compared with controls (risk ratio = 0.69 [0.37–1.29]), underscoring its favorable safety profile (Ko et al., 2021; Kim et al., 2023). Collectively, these findings identify SG as a well-tolerated and efficacious therapeutic option that expands the evidence base for polyherbal interventions in FD.

The meta-analysis results indicated that adverse events were significantly less common with SG than with prokinetic agents. This finding is particularly relevant in light of concerns regarding the safety of certain prokinetics, such as domperidone, which has been linked to cardiac adverse events—including QT interval prolongation and ventricular arrhythmias—as well as rare extrapyramidal adverse events (Reddymasu et al., 2007; van Noord et al., 2010; Leelakanok et al., 2016). In contrast, mosapride has demonstrated a favorable cardiac safety profile in clinical studies (Nachimuthu et al., 2012). While herbal medicines are generally considered to have a favorable safety profile, they are not entirely devoid of risk. Previous systematic reviews have reported that most adverse events associated with herbal preparations are typically transient and mild, such as mild allergic reactions or gastrointestinal discomfort, with serious adverse events being rare (Hu et al., 2020; Luo et al., 2022; Yao et al., 2022). Nevertheless, continuous pharmacovigilance and rigorous quality control remain important to ensure the safe use of herbal medicines in clinical practice (Hu et al., 2020; Yao et al., 2022).

While the included studies in this meta-analysis, which mostly involved short-term administration (4 weeks), reported a favorable safety profile for SG, the broader safety aspects, potential side effects, and herb–drug interactions associated with its constituent herbs warrant careful consideration. For instance, *Glycyrrhiza uralensis* Fisch. ex DC. [Fabaceae; *Glycyrrhizae Radix et Rhizoma*], a component of SG, contains glycyrrhizin, which can cause pseudoaldosteronism (leading to edema, hypokalemia, and hypertension) when consumed in high doses or for prolonged periods (Isbrucker and Burdock, 2006). Furthermore, interstitial pneumonitis, although rare, is a well-known serious adverse event associated with Sho-saiko-to (Sosiho-tang), one of the base formulas for SG; this risk is primarily reported in Japan and has been linked to factors such as long-term administration or co-administration with interferon (Lee et al., 2011). In terms of interactions, the glycyrrhizin from *Glycyrrhiza uralensis* Fisch. ex DC. [Fabaceae; *Glycyrrhizae Radix et Rhizoma*] may potentiate potassium loss when combined with diuretics such as thiazides or furosemide (Farese et al., 1991). Moreover, Sho-saiko-to (Sosiho-tang) was shown *in vivo* in humans to reduce CYP1A2 activity and show a tendency to lower CYP3A activity, potentially altering the metabolism of co-administered drugs (Saruwatari et al., 2003). Therefore, while short-term use of SG for FD appears relatively safe as indicated by our findings, clinicians should remain vigilant for these potential risks and interactions, especially in patients receiving long-term treatment or polypharmacy.

A major strength of this review lies in its comprehensive search strategy across multiple languages and databases, which minimized selection bias and captured a broad spectrum of evidence. Furthermore, the use of rigorous inclusion criteria, restricted to RCTs and monotherapy interventions, enhanced the internal validity of our findings. However, limitations related to the methodological quality of the included studies remain. Most studies lacked adequate blinding and allocation concealment, posing a high risk of bias according to GRADE criteria. Regarding FD, subjective outcomes (total effective rate, symptom scores) are highly susceptible to expectation and detection biases when comparing dissimilar interventions like decoctions and tablets. Inadequate allocation concealment further raises concerns about potential selection bias. To strengthen the evidence, future RCTs should prioritize methodological rigor, with particular emphasis on effective blinding. Potential strategies to enhance the methodology include formulating both interventions as identical tablets, administering both as decoctions using a high-fidelity placebo (matching taste, color, smell), or employing a double-dummy design. Robust randomization, allocation concealment, and blinding are essential to validate the promising yet methodologically limited evidence for SG. In addition, the limited number of included studies and participants, particularly for secondary outcomes, resulted in imprecision and a downgrading of evidence quality in the GRADE assessment. While this review provides the first quantitative synthesis for SG, the number of included studies (N = 12) is considerably smaller than that available for other formulas, such as RKT (N = 52) and BXT (N = 57) (Ko et al., 2021; Kim et al., 2023). This disparity in the volume of evidence means that the precision of our effect estimates may be lower, and direct comparisons should be made with caution. The use of total effective rate and TCM symptom scores, which are not internationally standardized, may limit generalizability to non-Asian populations. Similarly, since all studies were conducted in China, the findings may not be applicable to other countries or healthcare systems.

## 5 Conclusion

Our findings suggest that SG could be a valuable therapeutic option for FD, especially in patients intolerant of or unresponsive to conventional prokinetics. Given its complex and multifactorial pathogenesis, the multi-targeted nature of herbal formulas, such as SG, may be particularly advantageous in FD. Nevertheless, the moderate-to-very low certainty of evidence, due to methodological limitations, warrants cautious interpretation of the findings. Future research should focus on large, well-designed, double-blind, multicenter RCTs with standardized outcome measures and robust reporting. Long-term safety studies and mechanistic investigations are also needed.

## Data Availability

The original contributions presented in the study are included in the article/Supplementary Material, further inquiries can be directed to the corresponding author.

## Abbreviations

BXT, Banxia-xiexin-tang; CI, confidence interval; FD, Functional dyspepsia; RCT, randomized controlled trial; RKT, rikkunshito; SG, Sihogyeji-tang; TCM, traditional Chinese medicine; WM, Western medicine.
