# Supplementary material for: Effect of Sihogyeji-tang on functional dyspepsia: a systematic review and meta-analysis
Source: Front Pharmacol. 2025 Nov 19;16:1689132. doi: 10.3389/fphar.2025.1689132 (PMC12672881; doi:10.3389/fphar.2025.1689132)
Supplement: Supplementary file 1 [file Supplementaryfile1.docx]

Supplementary Material

# Appendix A: Detailed search strategies for English Databases

#1. Sihogyeji-tang [MeSH Terms]

#2. (Sihogyeji* OR shihogyeji* OR Shiho-Guizhi* OR Saikokeishito* OR Saiko-keishi-to* OR Chaihuguizhi* OR Chaihu Guizhi* OR Chai hu gui zhi* OR Modified Chaihuguizhi* OR Modified Chaihu Guizhi* OR Modified Chai hu gui zhi*) [tiab]

#3. #1 OR #2

#4. Functional Dyspepsia [MeSH Terms]

#5. (“Functional Dyspepsia” OR Dyspepsia OR “Non-ulcer dyspepsia” OR Indigestion OR Digest* OR Gastr* OR Postprandial OR Epigastric OR Gut OR Stomach OR Intestin*) [tiab]

#6. #4 OR #5

#7. #3 AND #6

# Appendix B: Detailed search strategies for the Chinese Database

#1. SU='柴胡桂枝'

#2. SU='功能性消化不良'

#3. SU='非溃疡性消化不良'

#4. SU='消化不良'

#5. SU='消化'

#6. #2 OR #3 OR #4 OR #5

#7. #1 AND #6
